# Characteristics of Sleep Patterns in Adolescents: Comparisons between Saudi Arabia and the UK

**DOI:** 10.3390/healthcare10081378

**Published:** 2022-07-25

**Authors:** Wasmiah Bin Eid, An An Lieu, Michelle Jin Yee Neoh, Suhail Mahmoud Al-Zoubi, Gianluca Esposito, Dagmara Dimitriou

**Affiliations:** 1 Sleep Education and Research Laboratory, UCL Institute of Education, 25 Woburn Square, London WC1H 0AA, UK; wasminah.bineid.14@alumni.ucl.ac.uk; 2 Psychology Program, School of Social Sciences, Nanyang Technological University, Singapore 639818, Singapore; alieu001@e.ntu.edu.sg (A.A.L.); michelle008@e.ntu.edu.sg (M.J.Y.N.); 3 Department of Psychology, Sultan Qaboos University, Muscat 123, Oman; smalzoubi@squ.edu.om; 4 Department of Psychology and Cognitive Science, University of Trento, 38068 Rovereto, Italy

**Keywords:** sleep, adolescents, social media use

## Abstract

There are concerns regarding sleep deprivation among adolescents, especially with mounting evidence for the importance of sleep during puberty, and its effects on health and families. The present study aimed to characterise sleep in typical development (TD) adolescents in Saudi Arabia, and compare their sleep profiles to TD adolescents in the UK, to evaluate sleep patterns in adolescents in Saudi Arabia, and to examine the relationship between sleep patterns and the use of social media in both groups. Findings from the current study reported a shorter sleep duration for the Saudi Arabia group than in previous studies and the UK group, which may be attributed to the lack of sleep hygiene practised in Saudi Arabia. Multiple analysis of variance results found significant differences in daytime sleepiness (*p* < 0.001) and a preference for morningness/eveningness (*p* < 0.001) between Saudia Arabia and UK adolescents. Statistically significant negative correlations (*p* > 0.05) between the duration of social media usage and sleep duration were found in both the Saudi Arabia and UK adolescents, where a lower number of sleep hours was observed with a higher duration of social media use. This study fills a gap in the research of sleep in Saudi Arabia in adolescents, and offers important insights on the comparison in sleep habits between Saudi Arabia and UK adolescents.

## 1. Introduction

Currently, there is ample evidence that the ‘need’ for sleep changes with age, and that the importance of sleep is particularly important during the development of the brain. Adolescence is often considered a sensitive period, due to many changes related to metabolic functioning, sleep health and cognitive functioning, as well as many socio-emotional changes. During this developmental period, parental control tends to decrease, and parent–child sleep synchrony comes to a complete end. This period is largely characterised by the freedom of an independent bedtime and morning routine, increased social independence, usage of online media, as well as a turbulence of hormonal changes (e.g., [1]).

Recently, sleep medical professionals and research scientists have expressed a growing concern that sleep deprivation among young people is reaching epidemic levels, labelling this phenomenon as insufficient sleep syndrome due to self-induced sleep restriction [2], which is a hidden health crisis. It is evident that insufficient sleep is a global challenge affecting not only individual mental and general health, but also adversely impacting families and societies as a whole. The peak onset age of many mental health disorders, such as depression and anxiety, occurs during adolescence (e.g., [3]). The detrimental impact of poor sleep has been echoed across many countries, and is linked to the top five leading causes of death in the USA, including cardiovascular disease, cerebrovascular disease, road accidents, diabetes and hypertension [4]. Sleep has also been associated with cognitive performance, specifically attention [5].

### 1.1. The Adolescent Model—“Perfect Storm”

Considering the interplay of environmental and biological factors with sleep, pioneering work carried out by Carskadon and her team [6] undoubtedly provided the most plausible model of sleep during adolescence. In their multilevel model, sleep delay was related to biological factors such as pubertal changes, which include the expression of hormones such as cortisol and melatonin. Psychosocial factors that affect sleep patterns and contribute to phase delay were related to adolescent autonomy in their social activities. Taken together, the bioregulatory changes and social pressures were labelled as a “perfect storm”, pushing sleep times later and consequently reducing sleep duration. Carskadon et al. [7] found no impact of a single night of restricted sleep on cognitive performance scores for nine children (aged 11 to 13.2 years) using the Wilkinson Addition test and the Williams Word Memory test. However, several studies reported that the impact of sleep deprivation on cognition may be related to the task difficulty. For example, Randazzo and colleagues [8] found that a single night of restricted sleep had a negative impact on tasks which required a relatively higher cognitive load (e.g., verbal creativity and abstract thinking), but not on tasks such as verbal or general learning, in a group of children aged 10 to 14 years old.

The model was largely based on very limited data available at that time. However, a large number of studies have been carried out since, and Crowley and her colleagues [9] have updated their model of sleep based on these latest findings. Several studies examining optimal sleep duration for cognitive functioning and emotional regulation concluded that the mean sleep duration required was around 9 to 9.25 h per night for adolescents [10,11]. However, a large number of studies conducted across countries reported that the total sleep duration decreases with age, with an average of 7 h of sleep per night spent during the adolescent period [12]. As noted earlier, these are worrisome reports, considering the importance of sleep in other domains.

Moreover, the negative impact of psychosocial factors was clearly presented in a study when sleep duration was measured in laboratory settings where environmental triggers were removed (social media and schooling). Sleep duration increased to 9.25 h, in comparison to their sleep duration of 7 h in a home environment. Using the Perfect Storm model as a framework, the model suggests that bioregulatory factors do not support the exclusive role of pubertal changes, but it is the interplay with psychosocial factors that creates a negative environment for sleep [6].

It is also important to note that the jury is still out on the impact of a single night of sleep reduction on someone’s cognitive or behavioural performance. The Perfect Storm Model has been primarily used in research studies focusing on school times and sleep hygiene practices. However, other factors that are unique to individual families, such as religious activities, diet and cultural norms are yet to be considered, in particular, in Saudi Arabia where cultural norms have a major influence over individuals.

### 1.2. Psychosocial Pressures

Previous research findings have estimated that around 60% of adolescents have significantly less sleep than recommended (e.g., [13,14]), which is a minimum of 9 h of sleep (as outlined in Section 1.1 The Adolescent Model—“Perfect Storm”). Yet, a number of studies have provided consistent findings on the relationship between poor sleep and academic performance [15,16,17,18]. Arguably, many adolescents are engaged in a heavy workload at school, which leads to a delayed bedtime or reduced sleep duration. This is particularly evident in Asian countries, where academic performance is a top priority for families, and young people are engaged in extracurricular studies in favour of sleep [19]. Several additional factors have been associated with delayed time to bed and shorter sleep duration, including engagement in social media [20,21,22]. Adolescents are often regarded as “digital natives” because part of their daily life involves the heavy use of technology, such as mobiles, TV, online gaming and computers at schools. In recent years, studies have found evidence of the negative association between social media use and sleep quality (e.g., [23,24,25,26]). For example, very high social media users in a UK sample were more likely to report late sleep onset and wake times, as well as trouble falling asleep after nighttime awakenings [23]. Longitudinal findings also indicated that stress associated with social media use showed a positive relation with sleep latency and daytime sleepiness [27]. In addition, several studies have also examined sleep and its association with the use of social media and their contribution to negative health issues, such as increased risk of obesity due to an increased intake of food and a reduction in physical activities [28]. A recent systematic review also found links between sleep quality, social media use and mental health in youth. Specifically, the review found significant associations between excessive social media use and poor mental health outcomes, as well as poor sleep quality [29]. Yet, much of the research has focused on examining sleep in university students or adults, rather than adolescents (e.g., [30,31,32,33]). However, in recent years, the adolescent period has gained some attention; in particular, the impact of sleep restriction and social media on cognition and health. Several recent studies are outlined below.

Dimitriou and colleagues [17] established that bedtimes and sleep duration were associated with academic performance. They also correlated factors such as stimulants, including coffee and electronic media, before bedtime as having a negative impact on sleep. Another study reported that shorter sleep duration was associated with lower academic achievement, increased risk-taking behaviour, and a negative impact on mental and physical health [21,34,35]. Shorter sleep duration was also associated with increased daytime sleepiness [36]. These studies provide evidence that sleep duration in adolescence is restricted, due to increased pressure to use social media, and academic workload.

Thus far, only one longitudinal study has examined adolescents’ use of social networking. Vernon et al. [37] found that the frequent use of social media predicted an increase in sleep problems in 874 Australian adolescents, which in turn increased psychopathology. Comparably, Arora et al. [38] and Espinoza and Juvonen [39] reported that adolescents who frequently use social network sites were more likely to lose one hour’s sleep from their overall sleep duration.

In a recent study, Nosetti et al. [40] examined the sleep habits of 972 Italian adolescents (aged from 13 to 19 year old), using the Italian version of the School Sleep Habits Survey, and gathered data on the use of technology. Similar to the previous studies, the authors showed that internet use was related to later bedtimes and a shorter sleep duration. Over 18% indicated that they had poor sleep, and almost 30% indicated that they had an insufficient amount of sleep, 38% had difficulty falling asleep, 20% had nighttime wakings and 67% had difficulties in waking up.

### 1.3. Characteristics of Adolescent Sleep in Saudi Arabia

Understanding sleep patterns in adolescents from Saudi Arabia has been explored in a limited number of studies identified to date, highlighting that sleep benefits are overlooked in Middle Eastern countries. Nasim et al. [41] assessed sleep deprivation in a nationally representative sample in Saudi Arabia, and found that sleep deprivation—less than 7 h of sleep a day—was common amongst adolescents; 46% of the sample reported being sleep deprived. In addition, three-quarters of the sample reported that they felt unrefreshed upon awakening.

The study by Al-Hazzaa and colleagues [42] evaluated the relationship between sleep hours and obesity, and between sleep hours and lifestyle factors in adolescents (aged 14 to 23 years old). The authors found a mean self-reported sleep duration of just over 7 h, with girls having a significantly longer sleep duration than boys. It was also shown that over 31% of participants had less than 7 h of sleep, including daytime napping. In contrast with previous findings (e.g., [43]), screen time, and more frequent snacking and unhealthy eating habits were found to be significantly related with better sleep (over 8 h).

In another study, Merdad et al. [44] examined sleep in high school students ranging from 14 to 23 years old. Although this study made use of commonly used and validated questionnaires, such as the Pittsburgh Sleep Quality Index, the Epworth Sleepiness Scale, the Perceived Stress Scale and academic performance, these sleep questionnaires were not validated for use with children. The authors reported an average sleep duration of 7 h, and a sleep onset delay of 2.8 h during the week. An interesting finding was reported in relation to disturbed sleep cycles with 1 in 10 students reporting sleeping during the day after school to stay up at night. Factors associated with sleep restriction included stress, napping and caffeine use.

Both studies showed that sleep duration and sleep habits in Saudi Arabian adolescents are poor, and that the sleep duration was shorter than recommended by the American Sleep Medicine Society and the National Sleep Foundation. However, a direct comparison of the data was not possible, as the Al-Hazzaa and colleagues [42] study used self-developed questionnaires, whereas Merdad et al. [44] used subjective adult sleep questionnaires.

### 1.4. The Current Study

The current study aimed to characterise sleep in typical development (TD) adolescents in Saudi Arabia, and to compare their sleep profiles to TD adolescents in the UK. Neither of the previous studies in Saudi Arabia used validated sleep questionnaires appropriate for this age group; hence, this will be the first study to utilise available and validated measures of sleep for adolescents. The aims of this study were: Firstly, to evaluate sleep characteristics patterns (based on sleep questionnaires) in TD adolescents aged between 12 and 18 years old, in both Saudi Arabia and the UK. Secondly, to examine the relationship between sleep patterns and the use of social media.

The two previous related studies provided mixed findings; however, it is hypothesised that the TD group in Saudi Arabia will have shorter nocturnal sleep, and more sleep disturbances than their TD peers in the UK. The use of social media will be correlated with sleep parameters; in particular, a shorter sleep duration.

## 2. Methods

### 2.1. Participants

A total of 76 participants were recruited for the Saudi Arabia sample (M_age_ = 14.20, SD_age_ = 1.78) and 58 participants were recruited for the UK sample (M_age_ = 14.35, SD_age_ = 1.80). The inclusion criteria for participation were: adolescents between 12 and 18 years, who habitually lived in the country: Saudi Arabia or UK, respectively. The exclusion criteria were: A diagnosis of comorbid medical or psychiatric disorders and conditions such as epilepsy, poorly controlled asthma or eczema, and cyanotic heart disease or severe respiratory disease, or if they were taking hypnotics or medication that were likely to interfere with their habitual sleep patterns. Based on the inclusion and exclusion criteria, the age of participants in the Saudi Arabia sample ranged from 12.00 to 17.50, with 35 (46%) males and 41 (54%) females. The ages of the participants in the UK sample ranged from 12.10 to 17.10, with 28 (48%) males and 30 (52%) females.

### 2.2. Procedures

The recruitment was conducted electronically, predominantly via email and social media platforms across the UK and Saudi Arabia, in accordance with GDPR and ethical guidelines. Legal guardians of potential participants contacted the researcher to receive detailed study information (for parents/caregivers and child participants) and hard copies of the questionnaire. School headteachers in Saudi Arabia were provided with study information, and meetings with parents and their children were arranged by the schools. For the UK recruitment, schools were contacted from the current database held by the team at the laboratory. Parents and adolescents were provided with a study information sheet, and the inclusion and exclusion criteria was explained to them, to ensure that the children met the inclusion criteria. All participants were given the opportunity to ask questions and to discuss the study. Written consents were obtained from all participants, and parental consent was obtained for those under the age of 18. They were also informed of their right to withdraw and withhold information from the study at any time without having to provide a reason. This study was approved by the UCL Institute of Education Research Ethics Committee (Approval number 16682/001).

### 2.3. Materials

All the questionnaires were translated according to the Guidelines for the Process of Cross-Cultural Adaptation of Self-Report Measures [45]. Materials used in the study—surveys, questionnaires and consent forms—were translated into Arabic by a certified professional with a degree in Arabic–English translation, and then reviewed by other native Arabic speakers with a PhD level education. The Arabic translation of the survey was then back-translated to English by another certified professional with a degree in Arabic–English translation.

### 2.4. School Sleep Habits Survey

The School Sleep Habits Survey (SSHS; [46]) was designed to measure adolescents’ sleeping related behaviour and daytime functioning. It includes 63 items, which consist of habitual weekday and weekend sleep profiles, daytime sleepiness, sleep/wake problem behaviours, circadian preference, a depressive mood scale and caffeine consumption. The reliability coefficients were Cronbach’s α(daytime sleepiness) = 0.70, Cronbach’s α(sleep/wake problem behaviours) = 0.75, Cronbach’s α(morningness/eveningness) = 0.75 and Cronbach’s α(depressive mood ) = 0.79. The questionnaire included questions regarding participant’s sleep habits over the past two weeks. In addition to background information and wake/sleep times, various items on the questionnaire made up four subscales.

#### 2.4.1. Daytime Sleepiness Scale

This scale asks participants whether they had struggled to remain awake in 10 different situations ranging from 1 (“no”) to 4 (“both struggled to stay awake and fallen asleep”). Scores on this scale can range from 10 to 40, where higher scores indicate greater sleepiness.

#### 2.4.2. Sleep/Wake Problem Behaviour Scale

In this scale, participants were asked how frequently 10 different behaviours applied to them, ranging from 5 (“everyday”) to 1 (“Never”). Scores on this scale ranged from 10 to 50, where higher scores indicated greater problem behaviour.

#### 2.4.3. Morningness/Eveningness (M/E) Scale

This scale is generated through responses to questions 47–56. Questions 47, 52 and 54 are scored from 5 to 1 (with the first response scored as 5 and the last as 1). Questions 48, 53 and 55 are scored from 1 to 4 (with the first response scored as 1 and the last as 4). Questions 49, 50, 51 and 56 are scored from 4 to 1 (with the first response scored as 4 and the last response scored as 1). Scores on the M/E scale range from 10 to 43, with the lower scores indicating an inclination towards the evening, and the higher scores indicating an inclination towards the morning.

#### 2.4.4. Depressive Mood Scale

In the Depressive Mood Scale [47], participants rated how frequently six items relating to a depressed mood applied to them, ranging from 1 (“not at all”) to 3 (“much”). Scores on this scale can range from 6 to 18, where higher scores indicate greater acute symptoms of depressed mood.

#### 2.4.5. Background Lifestyle Questionnaire

This questionnaire was designed to assess lifestyle habits and questions, including: smoking, the consumption of energy drinks, caffeine and alcohol; social media usage included television viewing, video games and the use of social apps [17]. However, due to cultural considerations, questions related to smoking and alcohol consumption were removed from the questionnaires in Saudi Arabia, and the focus was only on social media habits.

## 3. Results

The SSHS and background questionnaires were returned from all participants enrolled in the study. Summaries of each group’s results from the SSHS questionnaires are depicted in Table 1 and Table 2. Sleep variables (SSHS) and background lifestyle factors were analysed using partial Pearson’s Correlations. For all results, correlations greater than ±0.50 were taken as representing strong associations, and those between ±0.30 and ±0.50 were taken as moderate. Correlations below ±0.30 were considered weak and are not discussed in the body of the text [48].

### 3.1. Inferential Analyses: Group Comparisons of Sleep Variables

The total mean sleep time duration during the school day was 4 h and 40 min for adolescents in Saudi Arabia, which was significantly lower than the UK adolescents, who had a mean sleep duration of 5 h and 42 min (F(2,132) = 9.12, *p* < 0.002, R^2^ = 0.09). In contrast, the total sleep duration during the weekend increased in both groups; with a mean sleep duration of 7 h and 10 min for adolescents in Saudi Arabia, and a mean sleep duration of 9 h and 6 min in the UK adolescents. Again, there was a significantly shorter sleep duration in the Saudi Arabia group in comparison to the UK group (F(2,132) = 11.14, *p* < 0.001, R^2^ = 0.11).

**Figure 1:**
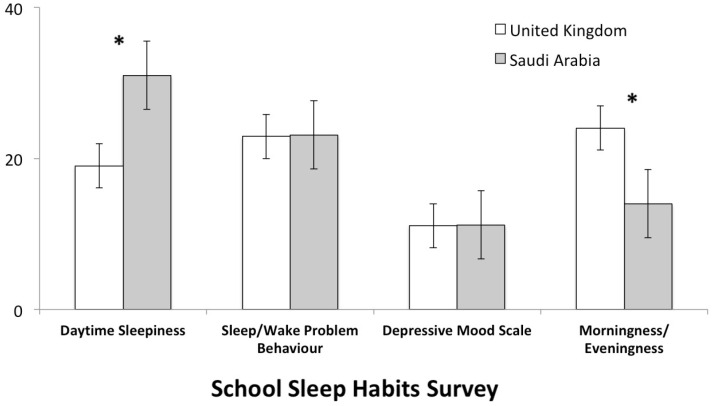
Mean percentage scores on the SSHS subscales. Daytime Sleepiness Scale: higher scores indicate daytime sleepiness; Sleep/Wake Problem Behaviour Scale: higher scores indicate greater problem behaviour. Morningness/Eveningness (M/E) Scale: lower scores indicate more eveningness and higher scores indicate more morningness. Depressive Mood Scale: higher scores indicate greater acute symptoms of depressed mood. * indicates a significant difference between the UK and Saudi Arabia groups.

Next, the groups were compared on each subscale of the School Sleep Habits Survey, using the MANOVA test. A significant difference between the Saudi Arabia and UK group was found on the daytime sleepiness scale, revealing that adolescents in Saudi Arabia had a higher level of daytime sleepiness compared to adolescents in the UK (F(2,132) = 16.52, *p* < 0.001, R^2^ = 0.18). On the scale of Morningness/Eveningness, the results indicated that adolescents in Saudi Arabia showed a significantly higher inclination for eveningness compared to the UK group (F(2,132) = 12.82, *p* < 0.001, R2 = 0.14). There were no significant differences between the two groups in terms of sleep/wake problem behaviour and depressive mood. There were also no significant differences in age or sex in relation to the scores on the SSHS (Figure 1).

### 3.2. Inferential Analyses: Correlations between Lifestyle Factors and Sleep Variables

Zero Order correlational analysis was carried out in relation to the lifestyle factors gathered in the background measures (energy drink, caffeine and alcohol consumption; social media usage included television viewing, video games and use of social applications) in order to examine the relationship between these factors and sleep duration. There was a significant correlation between the duration of social media usage and sleep duration for both the Saudi Arabia group (r = 0.34, *p* < 0.01) and the UK group (r = 0.38, *p* < 0.01), and these correlations were not statistically different in the two countries (Fisher *z* = *ns*). The relationship between social media use and sleep duration was such that the higher the duration of social media use, the lower the number of sleep hours. No other correlations were significant within and between the groups.

## 4. Discussion

The previous studies and the Perfect Storm proposed by Carskadon [6] indicated that adolescents are subjected to psychosocial and biological pressures that result in later bedtimes and shorter sleep duration. The main aim of this study was to evaluate sleep patterns in adolescents in Saudi Arabia who are influenced by different cultural and religious practices, in comparison to the adolescents in the UK. The secondary aim was to examine the relationship between sleep patterns and the use of social media in both groups. Findings from the current study were partially consistent with findings from previous studies conducted in Saudi Arabia outlined in the introduction in this chapter. The Saudi adolescent group in the present study reported a shorter sleep duration than in previous studies; however, this study also reported a much shorter sleep duration than in any other previous studies carried out in this young population. The mean duration was just over 4 h and 40 min, whereas previous studies reported mean sleep duration to be range from 7 to 7.2 h. However, Al-Hazzaa et al. [49] and Merdad et al. [44] also reported that over 30% of adolescents had a mean duration of less than 5 h of sleep per night.

RQ1: To evaluate sleep characteristics patterns (based on sleep questionnaires) in TD adolescents aged between 12 and 18 years old in both Saudi Arabia and the UK.

The current findings indicate that the Saudi Arabia adolescents in the sample are sleep deprived, as observed from the relatively short mean sleep duration of 4 h and 40 min. These results corroborate with recent findings in [41] and [50], which indicate that sleep deprivation is common among children and adolescents in Saudi Arabia. We suggest that there are three key factors that may possibly contribute to the sleep deprivation observed in the Saudi Arabia adolescents in this sample, namely self-induced sleep restriction, culturally induced sleep restriction, and society-induced sleep restrictions. The self-induced factors include an extensive use of social media platforms, cultural induced factors include a late social life and religious observations, and society-induced factors are related to early school times and long commuting times to schools, as well as a lack of societal awareness regarding healthy sleep.

The higher levels of daytime sleepiness experienced by adolescents in Saudi Arabia may support recent studies that request for later school start times [51,52]. Not only are the school start times much earlier in Saudi Arabia than in the UK or USA, but a longer duration is also needed for the commute to school, as the Saudi transport infrastructure is in its early stage of development. Saudi adolescents’ mean sleep duration was reported to be 4 h and 40 min which is drastically unhealthy and may lead to numerous undesired health issues. One possible way to increase sleep duration and to reduce daytime sleepiness would be later school start times. It is also interesting that although the current study used age-appropriate SSHS questionnaires, findings from the current study corroborate others suggesting that the shorter sleep duration seen in the UK sample is in line with the previous studies (e.g., [17]).

RQ 2: Examine the relationship between sleep patterns and the use of social media.

The results showed that social media use was correlated with a reduction in overall sleep duration, which was consistent with results in the existing literature demonstrating the negative association between social media use and sleep (see [53] for a review). Arora et al. [38] and Espinoza and Juvonen [39] also reported similar findings, with adolescents losing around an hour of sleep due to social media. Specifically, Espinoza and Juvonen [39] found that 37% of American adolescents reported losing one hour’s sleep due to the use of social networking sites. Arora et al. [38] reported similar findings, as adolescents who frequently use social network sites were more likely to lose one hour’s sleep from their overall sleep duration. The current findings showed even larger sleep restrictions in these groups, which cannot be fully explained from the data collected, although findings provide further support that social media has a negative effect on adolescent sleep duration, as it is associated with a reduction in the overall sleep duration in both countries (e.g., [21]). There are a number of mechanisms that may be at play in the association between media exposure and sleep [54]. It is possible that using social media before bed displaces the natural circadian rhythm overtime. An extra half an hour watching a program changes the circadian clock, which becomes entrained by the new sleep pattern. This has the potential for bedtimes to gradually become later. Stimulating television shows and even social media may serve to increase physiological arousal, making it more difficult to sleep, which can be observed from the effect of playing computer games before bedtime [55,56]. Lastly, the bright light that emanates from television and computer screens, and specifically the electromagnetic radiation from tablets and more advanced mobile telephones, may serve to suppress melatonin levels [57,58], the hormone responsible for readying the body for sleep.

## 5. Limitations

A limitation of the current study is the small sample size of participants who have lived in a large city. Due to the small sample size, results in the current study are limited in generalising to the entire Saudi Arabian population, and should be corroborated with future studies conducted on Saudi Arabia adolescent samples. Specific cultural and societal factors influencing sleep in adolescents should also be examined in future studies, in order to gain an insight into the cross-cultural differences in sleep. For example, differences in school start times and academic pressure between countries may be societal and cultural factors that may influence the sleep patterns of adolescents. Secondly, the data were collected using subjective sleep measures and subjective measures of lifestyle habits. This means that the measurements of sleep and lifestyle habits may not have been accurately and fairly analysed. Therefore, it would be advantageous to use objective sleep measures such as actigraphy across a number of days, as well as having a tracker of the social media usage. This will allow for more objective measurements of sleep and social media usage, to ensure that the results from the study are more accurately reported. Another interesting area for future investigation would be to examine sleep during the weeks of Ramadan, when society-induced changes occur as people fast from dawn to sunset. This may potentially affect sleep times and social media usage. Thirdly, data were not collected on pubertal maturation, which could either be subjectively assessed using the Tanner’s staging [59] or objectively assessed via the use of biomarkers such as testosterone and estrogen salivary concentrations. This can be beneficial in exploring the biological impact of hormones on adolescents’ sleep time. Lastly, the current study conducted a correlational analysis between social media use and sleep times in adolescents, and cannot be used to definitively establish a causal relationship between the two. In order to further strengthen the findings reported in the present study, future studies can employ a longitudinal design in examining the association between social media use and sleep patterns in children and adolescents across development.

## 6. Conclusions

Given that past research has highlighted the importance of sleep for adolescents, the findings in the present study regarding the short sleep duration observed in adolescents in both Saudi Arabia and UK strongly suggest that more attention should be given to promoting sleep hygiene in adolescents. The results in the current study also corroborate with the existing literature of the influence of social media use on sleep. Since a higher social media usage was significantly associated with reduced sleep, this suggests that targeting social media use in adolescents may be a possible avenue in promoting healthy sleep habits. Lastly, it is important to note that different sociocultural factors at play across countries may also contribute to sleep patterns in adolescents, and accounting for these differences in sociocultural contexts can be important for parents and clinical practitioners in promoting healthy sleep habits instead of directly adopting recommendations that were developed based on empirical research conducted in a different country.

## Figures and Tables

**Table 1:** Detailed summaries of the sleep patterns for TD adolescents in the UK, based on the scores derived from the School Sleep Habits Survey.

UK Sample (*N* = 58)	Minimum	Maximum	*Mean*	*SD*
Bedtime Weekdays (hh:ms)	21:00	02:03	23:37	0:46
Wake Up Weekdays (hh:ms)	06:02	07:10	6:30	0:19
Total Sleep Time Weekdays (hh:mm)	5:00	8:12	5:42	0:30
Bedtime Weekends (hh:ms)	1:30	4:31	2:09	0:47
Wake Up Weekends (hh:ms)	09:00	14:30	11:30	01:19
Total Sleep Time Weekends (hh:mm)	6:00	11:20	9:06	0:47
*Daytime Sleepiness (10–40)*	12	36	18	6.2
*Sleep/Wake Problem Behaviour (10–50)*	12	36	18	6.2
*Depressive Mood Scale (6–18)*	6	22	11	2.8
*Morningness/Eveningness (10–43)*	13	38	24	7.3

**Table 2:** Detailed summaries of the sleep patterns for TD adolescents in SA, based on the scores derived from the School Sleep Habits Survey.

SA Sample (*N* = 76)	Minimum	Maximum	*Mean*	*SD*
Bedtime Weekday (hh:ms)	23:20	01:40	23:50	00:42
Wake Up Weekdays (hh:ms)	05:03	05:50	05:30	00:17
Total Sleep Time Weekdays (hh:mm)	03:00	07:30	04:40	00:28
Bedtime Weekends	02:50	09:32	07:09	00:49
Wake Up Weekends	09:40	11:30	10:00	01:12
Total Sleep Time Weekends	05:30	09:30	07:10	00:41
*Daytime Sleepiness (10–40)*	21	36	31	4.2
*Sleep/Wake Problem Behaviour (10–50)*	14	44	22	6.8
*Depressive Mood Scale (6–18)*	7	21	11	2.3
*Morningness/Eveningness (10–43)*	12	18	14	4.3

## Data Availability

Data are not publicly accessible but can be made available on request to D.D.
